# Supplementary material for: Surface Plasmon-Enhanced Luminescence of CdSe/CdS Quantum Dots Film Based on Au Nanoshell Arrays
Source: Materials (Basel). 2019 Jan 24;12(3):362. doi: 10.3390/ma12030362 (PMC6384793; doi:10.3390/ma12030362)
Supplement: Supplementary file 1 [file materials-12-00362-s001.pdf]

Supplemental Information:

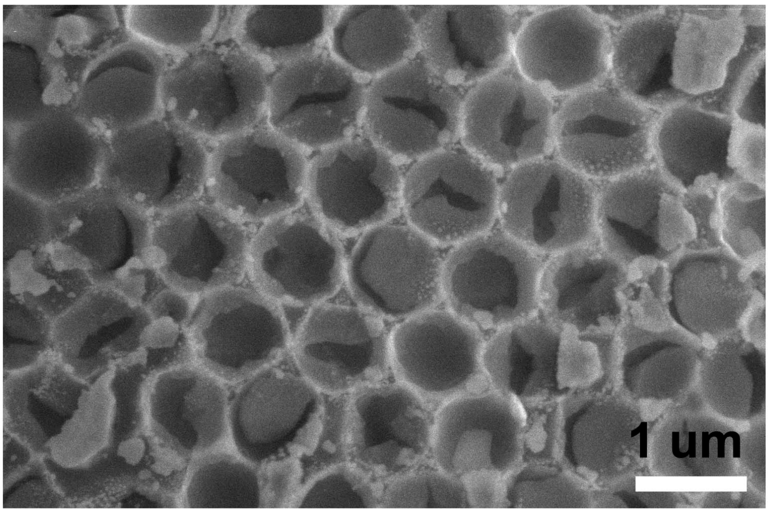

Figure S1. SEM of Au nanoshell arrays of 1000 nm after annealing for at 300 °C for 1 h.
